# Factors related to healthcare costs of road traffic accidents in Bucaramanga, Colombia

**DOI:** 10.11606/s1518-8787.2022056003299

**Published:** 2022-05-18

**Authors:** Raquel Rivera-Carvajal, Astrid Nathalia Páez-Esteban, Claudia Consuelo Torres-Contreras, Rafael Enrique Esquiaqui-Felipe, Nixon Ricardo González, Claudia Celmira Mejía-Muñoz

**Affiliations:** I Universidad de Santander. Facultad de Ciencias Medicas y de la Salud. Instituto Masira, Grupo Everest. Bucaramanga, Colombia; II Universidad de Santander. Facultad de Ciencias Medicas y de la Salud. Instituto Masira, Grupo de Salud Pública. Bucaramanga, Colombia; III Secretaria de Salud de Bucaramanga Observatorio Digital Municipal Bucaramanga Santander Secretaria de Salud de Bucaramanga. Observatorio Digital Municipal. Bucaramanga, Santander, Colombia; IV Dirección de Transito de Bucaramanga. División Estadísticas. Bucaramanga, Santander, Colombia; V Dirección de Transito de Bucaramanga. Subdirección Técnica. Bucaramanga, Santander, Colombia

**Keywords:** Accidents, Traffic, Health Expenditures, Factores de Riesgo, Estudios Transversales

## Abstract

**OBJECTIVE:**

To determine the factors related to overall healthcare costs of road traffic accidents in Bucaramanga, Colombia.

**METHODS:**

A descriptive cross-sectional study was conducted through the analysis of road traffic accident records that took place in 2019 in Bucaramanga, Colombia. Cost quartiles in dollars were compared using Pearson’s chi-squared and Fisher’s exact tests. Odds ratios were also calculated in logistic regression.

**RESULTS:**

3,150 road accidents were reported in 2019 involving 7,038 people, of which 812 had information related to healthcare costs in health care institutions. The median cost was 56.59 USD (RI = 29.35–140.15), average cost of 290.11 USD ± 731.22 (95%CI: 239.74–340.48). A higher possibility to be in the 4th quartile was found when persons were under 18 years of age (OR = 4.88; 95%CI: 1.30–18.32) or 46–60 years (OR = 3.66; 95%CI: 1.01–13.30), the type of vehicle involved is motorcycle (OR = 2.79; 95%CI: 1.25–6.24), bicycle (OR = 7.66; 95%CI: 2.70–21.68), having a head injury (OR = 4.50; 95%CI: 2.61–7.76) and hypothetical drunk driving (OR = 12.44; 95%CI: 2.01–76.87).

**CONCLUSIONS:**

Relevant factors in healthcare costs were riding a motorcycle or bicycle, having a head injury, being under 18 years of age or 46 to 60 years of age and hypothetical drunk driving. It is important to implement prevention measures based on identified factors to reduce road accident rate and therefore, its socioeconomic costs.

## INTRODUCTION

Traffic accidents or crashes represent a worldwide public health problem. The global burden of disease from road traffic crashes or accidents was 877.4 per 100,000 inhabitants (age-standardized rate) in 2016, being more frequent in Central and Eastern European countries^1 , 2^. Mortality due to this matter was 18.3 and years of life lost was 817.4 per 100,000 inhabitants^2^.

In Colombia, during 2019 the rate of traffic accidents was 1,214, with a mortality rate of 14.6 (95%CI: 11.1–18.6) per 100,000 inhabitants, the latter corresponds to 3.13% of the total deaths in the country, positioning it as the ninth cause of death. Mortality is higher in men (24 per 100,000 inhabitants) than in women (5.9 per 100,000 habitants); it is also more frequent in people over 70 years of age. Likewise, the years of life lost were 701.7 per 100,000 ihabitants, which places it in sixth place^3^.

Many factors can be associated with traffic accidents occurrence. A classification conducted in Mexico^4^ suggests that factors can be classified into three stages: before, during and after the event. In addition, factors can be grouped into individual factors (pedestrians, motorcyclists and/or drivers), vehicle factors (equipment and protection elements) and environmental factors (climatic factors and infrastructure), some of which can be opportunities for public health research and intervention^5^.

In addition, timely emergency care saves lives and reduces disabilities. It is estimated that if low-and middle-income countries had the same mortality rates from severe injuries that of high-income countries, 500,000 deaths caused by road crashes could be avoided each year^6^.

In this light, emergency care (pre-hospital, transport and services in healthcare provider institutions) should involve basic aspects such as ensuring access to emergency care, providing main structural components for prehospital care, implementing basic emergency service packages at each level of care, creating a national lead agency and evaluating the emergency care system^6^. The above is to reduce deaths and disabilities due to road traffic injuries, lower socioeconomic costs related to countries, families and individuals, have more efficient and effective use of existing healthcare resources at all levels of the system and expand emergency care capacity and resilience of the system to maintain service provision even during crashes with multiple victims.

Traffic accidents are estimated to take about 1 to 4% of the gross domestic product (GDP) in developing countries^7^. In Colombia, although healthcare service costs are covered by the Compulsory Traffic Accident Insurance (SOAT for its acronym in Spanish), which must be purchased for each vehicle, sometimes this insurance is not enough to cover these costs. Therefore, after exceeding insurance coverage limits, the bill is covered by health promoting entities. In Bogotá, healthcare costs of traffic accidents were estimated at $1,112,000 COP per patient ($278 USD), inpatient day at $1,200,000 and outpatient care at $247,400 COP^8^.

As previously described, this study was meant to determinate the different factors related with the total costs generated during heath care attentions for traffic accidents in Bucaramanga, Santander, Colombia, during 2019.

## METHODS

### Study Design and Population

A descriptive cross-sectional study was conducted by analyzing secondary data from traffic accident records in Bucaramanga in 2019 provided by the Traffic Directorate and the Municipal Digital Observatory of Bucaramanga using census sampling.

### Data Collection

Based on 7,038 records from people involved in 3,150 traffic accidents that took place in Bucaramanga during 2019 reported by the Traffic Directorate and the healthcare data reported by the Municipal Digital Observatory from healthcare provider institutions, it was possible to obtain costs for 812 people treated in multiple healthcare institutions around the city. Databases were extracted, cleaned-up and cross-referenced afterward ( Figure ).

**Figure:**
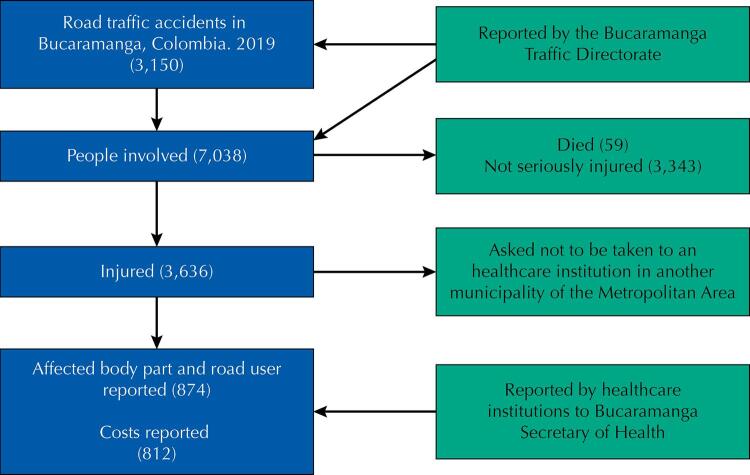
Process of obtaining information.

The dependent variable was direct healthcare costs (such as emergency care, in-patient care, surgical procedures, etc.) reported by healthcare institutions to the Bucaramanga Secretary of Health, including medical examination/care (by general practitioners, medical specialists and nursing), diagnostic aids (laboratory tests, X-rays, CT scans, MRI scans, etc.) and treatments (medication, surgical procedures.)

In addition, the following covariates were considered: identifier, gender (male, female), age (in years and categories of < 18, 18–25, 26–35, 36–45, 46–60 and > 60 years), type of vehicle involved (car, motorcycle, SUV, truck, bus), accident hypothesis (failure to obey traffic signals and rules, failure to keep safe distance, sharp turns, inexperienced driving, speeding, improper overtaking, etc.), place of occurrence, bearing a driving license and compulsory traffic accident insurance, location of healthcare institution, severity, date of occurrence, day of the week (Monday, Tuesday, Wednesday, Thursday, Friday, Saturday or Sunday), district, healthcare provider institution, surgery requirement, death, affected body part (head, neck, thorax, abdomen, upper limbs, lower limbs, several parts of the body, soft tissues, genitalia - of the International Classification of Diseases, 10th edition - ICD-10 based variable), diagnosis, road users and general healthcare costs in Colombian pesos.

### Data Analysis and Treatment

Variables were described by using proportions of qualitative variables, average, standard deviation, median and interquartile range according to the normal distribution of quantitative variables. The variable “costs” was converted into dollars (1 dollar = 4,000 COP according to the representative exchange rate on March 31, 2020 provided by the Financial Superintendence of Colombia). Quartiles were calculated afterward. Differences between quartiles were studied in relation to the variables age, sex, type of vehicle involved, day of the week, accident hypothesis, bearing a driving license and mandatory traffic accident insurance, affected body part, in addition to other traffic accident variables through Pearson’s chi-square and Fisher’s exact tests. Statistical significance was considered when the p-value was < 0.05.

Odds ratios were calculated by logistic regression, representing an increase in the possibility of being in the 4th quartile at a 95% confidence interval for the category of interest with respect to the reference category (assigned as “1”). As for the affected body part variable, the reference was several parts of the body when referring to injuries in several parts of the body. Data analysis was performed using the Stata 11.0 software (StataCorp College Station, United States.)

### Ethical Considerations

The project was approved under an internal call for proposals (code CIF0315-19) at the *Universidad de Santander* (UDES) by the University Ethics Committee (Minutes 019 of 2019) and the *Empresa Social del Estado Hospital Universitario de Santander* - HUS (Santander University Hospital) as recorded in Minutes 09 of 2019, classified as a minimal risk study according to the Colombian regulations, Resolution 8430 of 1993 by the Ministry of Health. Data was processed in accordance with the provisions of the Colombian Statutory Law 1581 of 2012 (Habeas Data Law), which establishes personal data protection regulations.

## RESULTS

According to the Municipal Traffic Directorate, 7,038 people were involved in 3,150 traffic accidents in 2019. Of these people, a high proportion was male at 88.48% (6,227) and 29.67% (2,088) were in the 26–35 age range. Median age was 34 years of age (RI = 26–46). In addition, 81.15% (5,711) had a driving license and 93.81% (6,602) had compulsory insurance. The most relevant accident hypotheses were failure to obey traffic signals and rules, failure to keep a safe distance, making a sharp turn, driver inexperience and speeding, respectively. Regarding the type of vehicle involved, cars and motorcycles were the most relevant. Saturdays and Tuesdays had the highest number of people injured in accidents ( Table 1 ). In addition, 51.66% (3,636) had some type of injury, 0.84% (59) died and 47.50% (3,343) had no severe injuries.

**Table 1:** Characteristics of people involved in road traffic accidents - Bucaramanga, Santander, Colombia, 2019.

Characteristics	%(n) (n = 7,038)
Gender	
Female	11.52 (811)
Male	88.48 (6,227)
Age Median (RI)	34 (26–46)
Age range	
< 18 years	5.36 (377)
18–25 years	19.37 (1,363)
26–35 years	29.67 (2,088)
36–45 years	19.51 (1,373)
46–60 years	19.34 (1,361)
> 60 years	6.76 (476)
Driving license	81.15 (5,711)
Mandatory insurance	93.81 (6,602)
Relevant accident hypothesis	
Failure to obey traffic signals and rules	25.69 (1,808)
Failure to keep a safe distance	21.81 (1,535)
Making sharp turns	3.59 (253)
Inexperienced driving	3.38 (238)
Speeding	2.71 (191)
Improper overtaking	2.02 (142)
Type of vehicle involved	
Car	39.41 (2,774)
Motorbike	34.27 (2,412)
SUV	11.41 (803)
Truck	3.42 (241)
Bus	3.00 (211)
Day of the week	
Monday	14.22 (1,001)
Tuesday	16.01 (1,127)
Wednesday	14.54 (1,023)
Thursday	14.45 (1,017)
Friday	14.48 (1,019)
Saturday	16.03 (1,128)
Sunday	10.27 (723)

Source: data from *Dirección de Tránsito Municipal de Bucaramanga, Santander, Colombia.*

According to the records provided by healthcare provider institutions in Bucaramanga, 874 people were admitted in 2019 from which it was possible to identify the most common body part affected in 665 people, being lower limbs, upper limbs, thorax and head the most affected parts, respectively ( Table 2 ).

**Table 2:** Type of injuries of people involved in road traffic accidents - Bucaramanga, Santander, Colombia, 2019.

Characteristics	%(n)
Severity reported by the traffic directorate	n = 7,038
Injured	51.66 (3,636)
Died	0.84 (59)
Not seriously injured	47.50 (3,343)
Assisted in healthcare institutions	27.12 (1,912)
Affected body part reported by the Secretary of Health	n = 874
Head	10.18 (89)
Neck	1.72 (15)
Thorax	11.44 (100)
Abdomen	0.69 (6)
Upper limbs	13.39 (117)
Lower limbs	18.31 (160)
Several body parts	18.76 (164)
Soft tissues	1.37 (12)
Genitalia	0.23 (2)
Other	23.91 (209)
Cost in USD (1 USD = 4,000 COP)	n = 812
Median (RI)	56.59 (29.35–140.15)
Median ± DE (95%CI)	290.11 ± 731.22 (239.74–340.48)

Source: data from *Dirección de Tránsito Municipal* and *Secretaría de Salud de Bucaramanga. *

Regarding costs, data from 812 people injured in traffic accidents helped to identify a median of $56.59 USD (RI = 29,35–140.15) and an average of $290.11 USD ± 731.22 (95%CI: 239.74–340.48).

Differences were observed in relation to the distribution of some sociodemographic variables among quartiles. Regarding gender, no significant differences were identified among quartiles. However, male gender had percentages higher than 83% in all quartiles. Regarding age, significant differences were identified (p-value of < 0.001) in which people under the age of 18 accounted for 10.84% (22) in the fourth quartile but lower than 7% in other quartiles. In addition, people between 46 and 60 years accounted for 13.79% (28) in the fourth quartile but lower than 10.50% in other quartiles. Thus, the ORs are significant for these two categories, compared to those over 60 years of age ( Table 3 ).

**Table 3:** Differences in cost quartile and OR to be in cost Q4 as per sociodemographic variables related to road accidents - Bucaramanga, Santander, Colombia, 2019.

Characteristic	Total	Q1	Q2	Q3	Q4	p	OR (95%CI)
			
		(0.53–29.35)	(29.36–56.55)	(56.62–139.9)	(140.31–6754)		
				
	(812) % (n)	(203) % (n)	(203) % (n)	(203) % (n)	(203) % (n)		
Gender						0.051	
Male	86.45 (702)	92.12 (187)	85.71 (174)	83.74 (170)	84.24 (171)		1
Female	13.55 (110)	7.88 (16)	14.29 (29)	16.26 (33)	15.76 (32)		0.78 (0.50–1.22)
Age range						**< 0.001**	
< 18 years	6.77 (55)	5.42 (11)	6.40 (13)	4.43 (9)	**10.84 (22)**		**4.88 (1.30–18.32)**
18–25 years	30.17 (245)	36.95 (75)	28.57 (58)	31.03 (63)	24.14 (49)		1.83 (0.52–6.37)
26–35 years	34.11 (277)	22.66 (46)	39.90 (81)	36.45 (74)	37.44 (76)		2.77 (0.80–9.53)
36–45 years	15.52 (126)	19.70 (40)	14.29 (29)	15.76 (32)	12.32 (25)		1.81 (0.50–6.54)
46–60 years	10.34 (84)	7.39 (15)	10.34 (21)	9.85 (20)	**13.79 (28)**		**3.66 (1.01–13.30)**
> 60 years	3.08 (25)	7.88 (16)	0.49 (1)	2.46 (5)	1.48 (3)		1
Driving license	75.86 (616)	81.28 (165)	78.82 (160)	78.33 (159)	**65.02 (132)**	**< 0.001**	**0.48 (0.33–0.68)**
Valid mandatory traffic accident insurance	92.12 (748)	93.10 (189)	93.10 (189)	95.07 (193)	**87.19 (177)**	**0.021**	**0.45 (0.26–0.76)**
Severity reported by the municipal traffic directorate						**0.044**	
Injured	92.24 (749)	91.13 (185)	89.66 (182)	93.60 (190)	94.58 (192)		1
Died	1.60 (13)	0.49 (1)	2.96 (6)	0.49 (1)	2.46 (5)		**0.39 (0.16–0.94)**
Not seriously injured	6.16 (50)	8.37 (17)	7.39 (15)	5.91 (12)	2.96 (6)		**0.34 (0.29–0.40)**
Accident hypothesis						0.086	
Failure to obey traffic signals and rules	39.16 (318)	35.47 (72)	44.83 (91)	37.93 (77)	38.42 (78)		1.51 (0.42–5.41)
Failure to keep a safe distance	10.22 (83)	11.82 (24)	11.33 (23)	9.85 (20)	7.88 (16)		1.11 (0.28–4.34)
Running a red light	4.19 (34)	2.96 (6)	1.97 (4)	5.42 (11)	6.40 (13)		2.88 (0.69–12.02)
Speeding	4.19 (34)	3.94 (8)	4.93 (10)	3.45 (7)	4.43 (9)		1.68 (0.38–7.24)
Hypothetical drunk driving	1.35 (11)	1.48 (3)	0	0	3.94 (8)		**12.44 (2.01–76.87)**
Inexperienced driving	2.71 (22)	4.93 (10)	0.49 (1)	2.46 (5)	2.96 (6)		1.75 (0.36–8.33)
Driving against traffic flow	1.85 (15)	1.48 (3)	2.46 (5)	0.99 (2)	2.46 (5)		2.33 (0.45–12.09)
Priority to the right	1.48 (12)	1.97 (4)	0.99 (2)	1.48 (3)	0.99 (3)		1.86 (0.23–14.64)
Improper overtaking	9.85 (20)	0.98 (2)	4.43 (9)	1.48 (3)	2.95 (6)		2.12 (0.41–10.87)
Overtaking on right	1.23 (10)	0.49 (1)	0.99 (2)	1.48 (3)	1.97 (4)		3.11 (0.52–18.38)
Overtaking by closing	2.09 (17)	2.96 (6)	1.97 (4)	1.97 (4)	1.48 (3)		1
Making sharp turns	5.54 (45)	9.85 (20)	1.97 (4)	0	0		0.85 (0.19–3.79)
Type of vehicle involved						0.064	
Motorbike	85.71 (696)	83.74 (170)	83.25 (169)	88.18 (179)	87.68 (178)		**2.79 (1.25–6.24)**
Car	7.88 (64)	10.84 (22)	11.33 (5)	5.91 (12)	3.45 (7)		1
Bicycle	4.06 (33)	2.96 (6)	2.46 (5)	2.96 (6)	7.88 (16)		**7.66 (2.70–21.68)**
SUV	1.48 (12)	1.97 (4)	1.48 (3)	1.48 (3)	0.99 (2)		1.62 (0.29–8.99)
Truck	0.12 (1)	0	0.49 (1)	0	0		-
Jeep	0.12 (1)	0	0	0.49 (1)	0		-
Dump truck	0.12 (1)	0	0	0.49 (1)	0		-
Minibus	0.25 (2)	0	0.49 (1)	0.49 (1)	0		-
Bus	0.25 (2)	0.49 (1)	0.49 (1)	0	0		-
Affected body part reported by the Secretary of Health						**< 0.001**	
Head	15.91 (95)	7.69 (11)	9.79 (14)	11.18 (17)	**33.33 (53)**		**4.50 (2.61–7.76)**
Thorax	14.91 (89)	12.59 (18)	16.78 (24)	16.45 (25)	13.84 (22)		1.17 (0.64–2.14)
Upper limbs	16.42 (98)	15.38 (22)	21.68 (31)	19.74 (30)	9.43 (15)		0.64 (0.33–1.24)
Lower limbs	24.46 (146)	20.28 (29)	32.17 (46)	25.66 (39)	**20.13 (32)**		1.00 (0.58–1.71)
Several body parts	28.31 (169)	44.06 (63)	19.58 (28)	26.97 (41)	23.27 (37)		1

Source: data from *Dirección de Tránsito Municipal* and *Secretaría de Salud de Bucaramanga* .

p-value: pearson’s chi-square and Fisher’s exact tests; OR: Odds Ratio; (95%CI): 95% confidence interval.

Regarding variables related to regulatory compliance, those who bear a driving license and mandatory insurance represented a lower percentage in Q4 than Q1-Q3. Here, ORs are protective and their confidence intervals are less than “1”. According to the severity reported by Municipal Traffic Directorate officials based on the people who attended healthcare institutions in Bucaramanga, 92.24% (749) were classified as injured, 1.60% (13) died and 6.16% (50) had no severe injuries. The ORs and CIs of the latter two categories are less than 1.

Regarding accident hypothesis, no statistically significant differences were found that could affect costs when comparing quartiles. However, ORs were 12.44 (95%CI: 2.01–76.87) for people with hypothetical drunk driving ( Table 3 ).

The main type of vehicle involved in patients attending healthcare institutions is motorcycle, having percentages higher than 83% in every quartile. Bicycles also ranked second having 7.88% in Q4. Thus, ORs identified were 2.79 (95%CI: 1.25– 6.24) and 7.66 (95%CI: 2.70–21.68) when comparing accidents involving cars.

Regarding body part affected, 33.33% (53) were head injuries having the highest proportion in Q4, followed by lower limb injuries at 20.13% (32). OR in head injuries was 4.50 (95%CI: 2.61–7.76) compared to the category involving other body parts ( Table 3 ).

## DISCUSSION

Only a few studies have reported characteristics and costs of road traffic injuries. In Colombia, few studies have described incidences, associated factors and injury characteristics in these events, reporting that motorcyclists suffer the most injuries^4 , 9^; most affected body parts are head and limbs^9^, from which cranioencephalic trauma is highly associated with mortality^4 , 10^; and disobeying traffic rules^4^ and reckless driving^10^ were found to be as risk factors.

In our study, many traffic accidents involved young men bearing a valid driving license but did not follow traffic signals/rules or speed, which is consistent with the findings reported by other authors^11^.

Regarding the type of vehicle involved, cars were found to be in the first place after motorcycles^14^. However, according to the data from those attending healthcare institutions, the main vehicle involved was motorcycle as same as in other studies^12 , 16^.

Regarding injuries, in our study the most common body parts affected were upper limbs, lower limbs and head, in contrast to a study conducted in Brazil in which head injuries were identified as the most common body part affected. However, only motorcycle accidents were analyzed in the latter^17^.

In addition, the median cost per traffic accident was $56 USD (RI = 29.35–140.15) and the average was 290.11 ± 731.22, (95%CI: 239.74–340.48) only for medical expenses derived from in-patient care, which could be considered low compared to a study conducted in Iran where the average cost is $9,024 USD^11^. However, our study did not include indirect costs and costs of lost productivity derived from accidents, which according to other studies have shown to be higher than those purely related to healthcare due to accidents^15 , 18 , 19^.

Thus, costs derived from traffic accidents may have a social and economic impact on the community. Therefore, it is important to adopt preventive measures aimed at reducing their occurrence. One of these measures is to take actions to reduce traffic congestion as some studies have found that high levels of congestion can increase accident probability as a result of having more cars on the road. However, it should be considered that accidents are also frequent in low levels of congestion as a result of speeding. The latter suggests the need to intervene in regulations and speed controls on the road^20^.

Regarding this finding, it is suggested to continue exploring the effects of traffic congestion on road accidents, given the results shown in this study, such as failure to yield the right of way, overtaking by encroaching, overtaking on the right and making sharp turns, could indicate a behavior resulting from rushing or haste after a period of vehicular traffic.

In our study, a statistically significant relationship was found between traffic accident costs and age, possession of a valid driving license and insurance, road accident severity based on the classification made by the traffic directorate and affected body parts, similar to that reported in a study conducted in Iran^11^. A statistically significant relationship was also observed between injury severity and cost increase, similar to that reported in a study in Haiti where patients injured in road accidents represented the highest cost averaging $1,220 USD^22^.

In Argentina, Brazil, Colombia and Mexico, cumulative GDP loss was estimated at 13.540 USD million between 2006 and 2015 due to frequent chronic noncommunicable diseases, heart disease, stroke and diabetes^23^. In Colombia, the *Universidad de los Andes* estimated traffic accident costs to be close to 1% of GDP between 2008 and 2010. The above included medical, human and administrative costs, as well as economic losses due to property damage. In 2016, traffic accident costs incurred by the Compulsory Traffic Accident Insurance and the Administrator of the Resources of the General System of Social Security in Health were estimated at 1.5 COP billion per year^24^.

Although our study did not find a statistically significant relationship between gender and costs, a higher number of accidents involved men, which could be considered a risk factor and focus of preventive measures in this population, as identified in Brazil^14^ where this condition was considered a factor for increased sequelae and therefore, increased costs due to loss of production capacity.

Study results showed the need to have a prevention focus on the most frequent factors identified in traffic accidents such as male gender, young age, motorcycle as vehicle type and the prevalence of some inappropriate driving behaviors, which are not related to having a valid driving license insurance documentation. This suggests that educational actions should be focused on generating collective awareness to change risk behaviors in accidents. It is worth noting that several countries are now implementing a point-based driving license^25^ as a way to monitor and re-educate drivers. This would reduce traffic accident expenses in the healthcare system and the community, enabling resource allocation to supply other basic needs of the population and thereby likely reducing the nation’s economic burden^22^.

This study had some recognized limitations such as the lack of information from healthcare institutions located in other municipalities of the metropolitan area (Floridablanca, Piedecuesta and Girón). In addition, as for the ICD-10 data reported by healthcare institutions, 209 records did not provide any information on the body part that was specifically affected, assigning ICD-10 to the road user (pedestrian, cyclist, motorcyclist, falling.)

In addition, generated costs may be lower given that human and technological resources for healthcare may be lower than those of developed countries. In addition, in future studies it will be worth linking variables such as alcohol intake, road conditions where traffic accidents occurred, use of personal protection, complications due to surgical procedures and analysis of indirect costs such as loss of production capacity due to sequelae after accidents, thus providing a broader view for budget allocation and rates foreseen by insurance companies to cover these events, as suggested in other studies^19 , 26^, besides breaking down costs of direct medical care by professionals, medications, transportation, diagnostic aids and devices^15 , 19^. In addition, analyzing the quality of motorcycles, personal protective equipment and their proper use is highly suggested.

Finally, a considerable increase in traffic accident costs was identified when the type of vehicle involved is motorcycle or bicycle, the age of the injured person is under 18 years of age or between 46 and 60 years of age, affected body part is head and accident hypothesis is drunk driving. In contrast, a decrease in traffic accident costs was identified when the driver complied with bearing a regulatory driving license and mandatory accident insurance policy. Identifying mechanisms to reduce these types of events is highly relevant as traffic accidents can be preventable, measures can be implemented to reduce their severity and therefore, their costs and consequences for society.
