# Supplementary material for: Factors related to healthcare costs of road traffic accidents in Bucaramanga, Colombia
Source: Rev Saude Publica. 2022 May 18;56:47. doi: 10.11606/s1518-8787.2022056003299 (PMC9165636; doi:10.11606/s1518-8787.2022056003299)
Supplement: Translation

# Translation: Factores relacionados con costos por atención médica de siniestros de tránsito en Bucaramanga, Colombia

**Authors:** Raquel Rivera-Carvajal, Astrid Nathalia Páez-Esteban, Claudia Consuelo Torres-Contreras, Rafael Enrique Esquiaqui-Felipe, Nixon Ricardo González, Claudia Celmira Mejía-Muñoz

**Affiliations:** I Universidad de Santander. Facultad de Ciencias Medicas y de la Salud. Instituto Masira, Grupo Everest. Bucaramanga, Colombia; II Universidad de Santander. Facultad de Ciencias Medicas y de la Salud. Instituto Masira, Grupo de Salud Pública. Bucaramanga, Colombia; III Secretaria de Salud de Bucaramanga. Observatorio Digital Municipal. Bucaramanga, Santander, Colombia; IV Dirección de Transito de Bucaramanga. División Estadísticas. Bucaramanga, Santander, Colombia; V Dirección de Transito de Bucaramanga. Subdirección Técnica. Bucaramanga, Santander, Colombia

**Keywords:** Accidentes de Tránsito, Gastos en Salud, Risk Factors, Cross-Sectional Studies

## Abstract

**OBJETIVO:**

Determinar los factores relacionados con los costos generales durante la atención en salud de siniestros de tránsito en Bucaramanga, Colombia.

**MÉTODOS:**

Estudio descriptivo, transversal, con análisis de registros de siniestros de tránsito durante 2019 en Bucaramanga, Colombia. Se realizó una comparación entre cuartiles de los costos en dólares con pruebas estadísticas de Chi-cuadrado de Pearson y exacta de Fisher. También, se calcularon *odds ratio* en regresión logística.

**RESULTADOS:**

En 2019 se registraron 3.150 siniestros, con 7.038 personas involucradas, de los cuales 812 tenían información de costos generados por atención en instituciones de salud. La mediana de costos fue US$ 56,59 (RI = 29,35–140,15), promedio de US$ 290,11 ± 731,22 (IC95% 239,74–340,48). Se observó mayor posibilidad para estar en cuartil 4 de los costos si la persona tenía menos de 18 años (OR = 4,88; IC95% 1,30–18,32) o entre los 46–60 años de edad (OR = 3,66; IC95% 1,01–13,30), cuando el tipo de vehículo es la motocicleta (OR = 2,79; IC95% 1,25–6,24) y la bicicleta (OR = 7,66; IC95% 2,70–21,68), si la lesión ocurre en la cabeza (OR = 4,50; IC95% 2,61–7,76) y si la hipótesis está relacionada con embriaguez (OR = 12,44; IC95% 2,01–76,87).

**CONCLUSIÓN:**

Los factores relevantes en los costos fueron la motocicleta, bicicleta, lesión en la cabeza, ser menor de 18 años o tener entre los 46 años y los 60 años y embriaguez como hipótesis. Es relevante implementar medidas de prevención según los factores identificados para disminuir la accidentalidad y, por ende, sus costos socioeconómicos.

## INTRODUCCIÓN

Los accidentes o siniestros de tránsito representan un problema de salud pública mundial. En el 2016, la carga global de la enfermedad por siniestros de tránsito fue de 877,4 por cada 100.000 habitantes (tasa estandarizada por edad), siendo más frecuente en los países de Europa central y oriental^1 , 2^. La mortalidad por esta problemática fue de 18,3, y los años de vida perdidos fueron 817,4 por cada 100.000 habitantes^2^.

En Colombia, durante el año 2019 la tasa de siniestros de tránsito fue de 1.214, con tasa de mortalidad de 14,6 (IC95% 11,1–18,6) por 100.000 habitantes, esta última corresponde al 3,13% del total de muertes en el país, posicionándose en la novena causa de muerte. La mortalidad es mayor en hombres con 24 por 100.000 hab. que en mujeres 5,9 por 100.000 hab.; también, es más frecuente en personas mayores de 70 años de edad. Asimismo, los años de vida perdidos fueron 701,7 por 100.000 habitantes, lo cual lo ubica en el sexto lugar^3^.

Son múltiples los factores que pueden asociarse con el desarrollo de los siniestros de tránsito. En una clasificación realizada en México^4^, se plantea que se pueden subdividir en tres momentos: antes, durante y posterior al evento. Además, los factores se agrupan en individuales (características del peatón, motociclista y/o conductor), del vehículo (elementos de equipamiento y protección) y del ambiente (factores climáticos e infraestructura), algunos de estos factores son oportunidades de investigación e intervención en salud pública^5^.

Por otro lado, la atención oportuna de emergencias salva vidas y reduce las discapacidades. Se estima que si los países de medios y bajos ingresos tuvieran las mismas tasas de letalidad derivadas de traumatismos graves que los países de ingresos altos, se podrían evitar cada año 500.000 defunciones causadas por accidentes de tránsito^6^.

En este sentido, la atención de las emergencias (prehospitalaria, transporte y los servicios en las instituciones prestadoras de servicios de salud) debe involucrar los siguientes aspectos básicos: garantizar el acceso a la atención de emergencias; garantizar los principales componentes estructurales de la atención prehospitalaria; establecer un paquete básico de servicios médicos de emergencias para cada nivel de atención; establecer un organismo director a escala nacional; y realizar evaluación del sistema de atención de emergencias^6^. Esto permite reducir las defunciones y discapacidades debidas a traumatismos por siniestros de tránsito, disminuir los costos socioeconómicos vinculados para los países, las familias y las personas, uso más eficiente y eficaz de los recursos de atención sanitaria existentes en todos los niveles del sistema y mayor capacidad de atención de emergencia y resiliencia del sistema para mantener la prestación del servicio aun en casos de accidentes con múltiples víctimas.

Se estima que los siniestros de tránsito consumen entre el 1% al 4% del producto interno bruto (PIB) en países en desarrollo^7^. En Colombia, los gastos de la atención de servicios de salud son asumidos por el Seguro Obligatorio para Accidente de Tránsito (SOAT), el cual es adquirido para cada automotor, en ocasiones este rubro no es suficiente para cubrirlos, por lo tanto, después de pasar ese límite, la cuenta es asumida por las Entidades Promotoras de Salud. Los costos de la atención de los accidentes de tránsito en Bogotá en 2013 fueron estimados en promedio por paciente en $1.112.000 pesos colombianos (en dólares US$ 278), el día de hospitalización en $1.200.000 y pacientes con atención ambulatoria en $247.400^8^.

Por lo anteriormente descrito, en este estudio se pretende determinar los factores relacionados con los costos totales generados durante la atención en salud de los siniestros de tránsito en Bucaramanga, Santander, Colombia, durante el 2019.

## MÉTODO

### Diseño del Estudio y Población

Este es un estudio descriptivo, transversal, con análisis de datos secundarios provenientes de los registros de accidentes de tránsito de Bucaramanga en 2019, proporcionados por la Dirección de Tránsito y el Observatorio Digital Municipal de Bucaramanga, muestreo tipo censo.

### Recolección de Datos

Con base en los 7.038 registros de personas involucrados de un total de 3.150 siniestros de tránsito ocurridos en Bucaramanga en 2019 reportados por la Dirección de Tránsito y la información de atención en salud del Observatorio Digital Municipal de Bucaramanga a partir de las diferentes instituciones prestadoras de salud (IPS), se obtuvo información de costos de 812 personas atendidas en las IPS de la ciudad; posteriormente, se realizó la extracción, depuración y cruzamiento de las bases de datos ( Figura ).

**Figura:** Proceso de obtención de la información.

IPS: institución prestadora de salud.

La variable dependiente fue costos directos derivados de la atención en salud (urgencias, hospitalización, quirófanos), que las IPS reportaron a la Secretaría de Salud de Bucaramanga que incluyen la valoración/atención (médico general, especialista y atención de enfermería), las ayudas diagnosticas (laboratorios, rayos X, tomografías, resonancias, etc.) y tratamientos instaurados (medicamentos, procedimientos quirúrgicos).

Además, se tuvieron en cuenta las siguientes covariables: identificador, genero (femenino, masculino), edad (en años y en categorías de < 18 años, 18–25, 26–35, 36–45, 46–60 y > 60 años), tipo de vehículo involucrado (automóvil, motocicleta, camioneta, camión, bus), hipótesis del accidente (desobedecer señales y normas de tránsito, no mantener la distancia segura, girar bruscamente, impericia en el manejo, exceso de velocidad, adelantar invadiendo el carril), lugar de ocurrencia, porte de licencia, porte del seguro obligatorio de accidente de tránsito, sitio de atención en salud, gravedad, fecha de ocurrencia, día de la semana (lunes, martes, miércoles, jueves, viernes, sábado o domingo), comuna, instituciones prestadoras de servicios de salud, requerimiento de cirugía, muerte, parte del cuerpo afectada (cabeza, cuello, tórax, abdomen, miembros superiores, miembros inferiores, diferentes partes del cuerpo, tejidos blandos, genitales, esta variable organizada con base en los códigos de la Clasificación Internacional de Enfermedades, 10.ª edición – CIE-10), diagnóstico, actor vial y costo general de la atención en salud en pesos colombianos.

### Análisis y Tratamiento de los Datos

La descripción de las variables se realizó por medio de proporciones para variables cualitativas; promedios, desviación estándar, mediana y rango intercuartílico según la distribución normal o no de las variables cuantitativas. La variable costos se transformó a dólares, 4.000 pesos colombianos equivalen a 1 dólar según la tasa representativa del mercado a 31 de marzo del 2020 de la Superintendencia Financiera de Colombia, posteriormente, se calcularon sus cuartiles. Se exploraron diferencias entre los cuartiles con relación a las variables edad, sexo, tipo de vehículo involucrado, día de la semana, hipótesis de accidente, porte de licencia de conducción, porte de seguro obligatorio de accidentes de tránsito, parte del cuerpo afectada, además de otras variables del accidente de tránsito, a través de las pruebas estadísticas de chi-cuadrado de Pearson y exacta de Fisher. Se consideró significancia estadísticamente significativa cuando el valor de p es < 0,05.

También, se calcularon *odds ratio* (OR) con sus respectivos intervalos de confianza del 95% mediante regresión logística. Los OR representan la razón de posibilidades de estar en el último cuartil (Q4) de costos para la categoría de interés respecto a la categoría de referencia (se representa con el número “1”) de la variable evaluada. Para la variable parte del cuerpo afectada la categoría de referencia fue diferentes partes del cuerpo, la cual fue construida cuando los participantes referían lesiones en varias partes del cuerpo. El análisis de datos fue realizado con el software Stata 11.0 (StataCorp, College Station, Estados Unidos).

### Aspectos Éticos

El proyecto fue aprobado por la convocatoria interna de la Universidad de Santander (UDES) con el código CIF0315-19, por el Comité de Bioética de la Universidad de Santander (Acta 019 de 2019) y de la Empresa Social del Estado Hospital Universitario de Santander (HUS) (Acta 09 de 2019). El estudio fue catalogado como riesgo mínimo según la normatividad colombiana, Resolución 8430 de 1993 del Ministerio de Salud. El manejo de los datos se realizó conforme a lo estipulado por la Ley Estatutaria 1581 de 2012, Habeas Data de Colombia, que establece la protección de los datos personales.

## RESULTADOS

Según la Dirección de Tránsito Municipal, hubo 7.038 personas involucradas en 3.150 siniestros de tránsito durante 2019. De las personas involucradas, la mayor proporción corresponden al género masculino con el 88,48% (6.227) y rango de edad entre 26 y 35 años el 29,67% (2.088); mediana de edad de 34 años (RI = 26–46). Además, se tiene registro de que portaban la licencia de conducir el 81,15% (5.711) y el seguro obligatorio el 93,81% (6.602). Las hipótesis de accidentes más relevantes fueron desobedecer señales y normas de tránsito, no mantener la distancia segura, girar bruscamente, impericia al manejo y exceso de velocidad, en su orden. Con relación al tipo de vehículo involucrado, los más relevantes fueron automóvil y motocicleta. Los días sábado y martes fueron los que presentaron mayor número de personas lesionadas por estos eventos ( Tabla 1 ). Asimismo, el 51,66% (3.636) presentaron algún tipo de lesión, el 0,84% (59) fallecieron y el 47,50% (3.343) no tuvieron gravedad o lesión.

**Tabla 1:** Características de personas involucradas en siniestros de tránsito en Bucaramanga, Santander, Colombia, 2019.

Características	% (n) (n = 7.038)
Género	
Femenino	11,52 (811)
Masculino	88,48 (6.227)
Edad. Mediana (RI)	34 (26–46)
Rango de edad	
< 18 años	5,36 (377)
18–25 años	19,37 (1.363)
26–35 años	29,67 (2.088)
36–45 años	19,51 (1.373)
46–60 años	19,34 (1.361)
> 60 años	6,76 (476)
Porta licencia	81,15 (5.711)
Porta seguro obligatorio	93,81 (6.602)
Hipótesis relevantes del siniestro	
Desobedece señales y normas de tránsito	25,69 (1.808)
No mantiene la distancia segura	21,81 (1.535)
Girar bruscamente	3,59 (253)
Impericia en el manejo	3,38 (238)
Exceso de velocidad	2,71 (191)
Adelantar invadiendo el carril	2,02 (142)
Tipo de vehículo involucrado	
Automóvil	39,41 (2.774)
Motocicleta	34,27 (2.412)
Camioneta	11,41 (803)
Camión	3,42 (241)
Bus	3,00 (211)
Día de la semana	
Lunes	14,22 (1.001)
Martes	16,01 (1.127)
Miércoles	14,54 (1.023)
Jueves	14,45 (1.017)
Viernes	14,48 (1.019)
Sábado	16,03 (1.128)
Domingo	10,27 (723)

Fuente: datos de la Dirección de Tránsito de Bucaramanga, Santander, Colombia.

De acuerdo a los registros proporcionados por las IPS de Bucaramanga en 2019, ingresaron 874 personas a estas instituciones, de las cuales se obtuvo información de la parte del cuerpo afectada de 665 personas, siendo las más frecuentes en su orden: miembros inferiores, miembros superiores, tórax y cabeza ( Tabla 2 ).

**Tabla 2:** Tipo de lesiones en siniestros de tránsito en Bucaramanga, Santander, Colombia, 2019.

Características	% (n)
Gravedad dato Dirección de Tránsito Municipal	n = 7.038
Herido	51,66 (3.636)
Muerto	0,84 (59)
Sin gravedad	47,50 (3.343)
Atención en IPS	27,12 (1.912)
Parte del cuerpo afectada, dato Secretaría de Salud	n = 874
Cabeza	10,18 (89)
Cuello	1,72 (15)
Tórax	11,44 (100)
Abdomen	0,69 (6)
Miembros superiores	13,39 (117)
Miembros inferiores	18,31 (160)
Diferentes partes del cuerpo	18,76 (164)
Tejidos blandos	1,37 (12)
Genitales	0,23 (2)
Otros	23,91 (209)
Costos U$ dólares (4.000 pesos)	n = 812
Mediana (RI)	56,59 (29,35–140,15)
Media ± DE (IC95%)	290,11 ± 731,22 (239,74–340,48)

Fuente: datos de la Dirección de Tránsito Municipal y la Secretaría de Salud de Bucaramanga.

IPS: institución prestadora de salud.

Con relación a los costos, se obtuvo información de 812 personas lesionadas por siniestros de tránsito y se identificó una mediana de US$ 56,59 (RI = 29,35–140,15) y un promedio de US$ 290,11 ± 731,22 (IC95% 239,74–340,48).

Se observaron diferencias en la distribución de algunas variables sociodemográficas entre los cuartiles. No se identificaron diferencias significativas entre los cuartiles en relación al género, sin embargo, en todos los cuartiles, el género masculino tiene porcentajes mayores al 83%. En cuanto a la edad, se identificaron diferencias significativas (valor p < 0,001); en el cuarto cuartil las personas menores de 18 años representan el 10,84% (22) y en los otros cuartiles representan porcentajes inferiores al 7%. Además, en el cuarto cuartil las personas de 46 a 60 años corresponden al 13,79% (28) y en los otros cuartiles los porcentajes son menores al 10,50%. De esta forma, los OR son significativos para estas dos categorías, en comparación con los mayores de 60 años ( Tabla 3 ).

**Tabla 3:** Diferencias en el cuartil de costos y OR para estar en el Q4 de los costos según variables sociodemográficas y relacionadas con el siniestro en Bucaramanga, Santander, Colombia, 2019.

Característica	Total	Q1	Q2	Q3	Q4	p	OR (IC95%)
			
		(0,53–29,35)	(29,36–56,55)	(56,62–139,9)	(140,31–6.754)		
				
	(812) %(n)	(203) %(n)	(203) %(n)	(203) %(n)	(203) %(n)		
Género						0,051	
Masculino	86,45 (702)	92,12 (187)	85,71 (174)	83,74 (170)	84,24 (171)		1
Femenino	13,55 (110)	7,88 (16)	14,29 (29)	16,26 (33)	15,76 (32)		0,78 (0,50–1,22)
Rango de edad						**< 0,001**	
< 18 años	6,77 (55)	5,42 (11)	6,40 (13)	4,43 (9)	**10,84 (22)**		**4,88 (1,30–18,32)**
18–25 años	30,17 (245)	36,95 (75)	28,57 (58)	31,03 (63)	24,14 (49)		1,83 (0,52–6,37)
26–35 años	34,11 (277)	22,66 (46)	39,90 (81)	36,45 (74)	37,44 (76)		2,77 (0,80–9,53)
36–45 años	15,52 (126)	19,70 (40)	14,29 (29)	15,76 (32)	12,32 (25)		1,81 (0,50–6,54)
46–60 años	10,34 (84)	7,39 (15)	10,34 (21)	9,85 (20)	**13,79 (28)**		**3,66 (1,01–13,30)**
> 60 años	3,08 (25)	7,88 (16)	0,49 (1)	2,46 (5)	1,48 (3)		1
Porta licencia	75,86 (616)	81,28 (165)	78,82 (160)	78,33 (159)	**65,02 (132)**	**< 0,001**	**0,48 (0,33–0,68)**
Seguro obligatorio de accidente al día	92,12 (748)	93,10 (189)	93,10 (189)	95,07 (193)	**87,19 (177)**	**0,021**	**0,45 (0,26–0,76)**
Gravedad según dato Dirección de Tránsito Municipal						**0,044**	
Herido	92,24 (749)	91,13 (185)	89,66 (182)	93,60 (190)	94,58 (192)		1
Muerto	1,60 (13)	0,49 (1)	2,96 (6)	0,49 (1)	2,46 (5)		**0,39 (0,16–0,94)**
Sin gravedad	6,16 (50)	8,37 (17)	7,39 (15)	5,91 (12)	2,96 (6)		**0,34 (0,29–0,40)**
Hipótesis del accidente						0,086	
Desobedecer señales o normas de tránsito	39,16 (318)	35,47 (72)	44,83 (91)	37,93 (77)	38,42 (78)		1,51 (0,42–5,41)
No mantener la distancia	10,22 (83)	11,82 (24)	11,33 (23)	9,85 (20)	7,88 (16)		1,11 (0,28–4,34)
Semáforo rojo	4,19 (34)	2,96 (6)	1,97 (4)	5,42 (11)	6,40 (13)		2,88 (0,69–12,02)
Exceso de velocidad	4,19 (34)	3,94 (8)	4,93 (10)	3,45 (7)	4,43 (9)		1,68 (0,38–7,24)
Embriaguez aparente	1,35 (11)	1,48 (3)	0	0	**3,94 (8)**		**12,44 (2,01–76,87)**
Impericia en el manejo	2,71 (22)	4,93 (10)	0,49 (1)	2,46 (5)	2,96 (6)		1,75 (0,36–8,33)
Transitar contravía	1,85 (15)	1,48 (3)	2,46 (5)	0,99 (2)	2,46 (5)		2,33 (0,45–12,09)
No respetar prelación	1,48 (12)	1,97 (4)	0,99 (2)	1,48 (3)	0,99 (3)		1,86 (0,23–14,64)
Adelantar invadiendo	9,85 (20)	0,98 (2)	4,43 (9)	1,48 (3)	2,95 (6)		2,12 (0,41–10,87)
Adelantar por la derecha	1,23 (10)	0,49 (1)	0,99 (2)	1,48 (3)	1,97 (4)		3,11 (0,52–18,38)
Adelantar cerrando	2,09 (17)	2,96 (6)	1,97 (4)	1,97 (4)	1,48 (3)		1
Girar bruscamente	5,54 (45)	9,85 (20)	1,97 (4)	0	0		0,85 (0,19–3,79)
Tipo de vehículo involucrado						0,064	
Motocicleta	85,71 (696)	83,74 (170)	83,25 (169)	88,18 (179)	**87,68 (178)**		**2,79 (1,25–6,24)**
Automóvil	7,88 (64)	10,84 (22)	11,33 (5)	5,91 (12)	3,45 (7)		1
Bicicleta	4,06 (33)	2,96 (6)	2,46 (5)	2,96 (6)	**7,88 (16)**		**7,66 (2,70–21,68)**
Camioneta	1,48 (12)	1,97 (4)	1,48 (3)	1,48 (3)	0,99 (2)		1,62 (0,29–8,99)
Camión	0,12 (1)	0	0,49 (1)	0	0		-
Campero	0,12 (1)	0	0	0,49 (1)	0		-
Volqueta	0,12 (1)	0	0	0,49 (1)	0		-
Buseta	0,25 (2)	0	0,49 (1)	0,49 (1)	0		-
Bus	0,25 (2)	0,49 (1)	0,49 (1)	0	0		-
Parte del cuerpo afectada, dato Secretaría de Salud						**< 0,001**	
Cabeza	15,91 (95)	7,69 (11)	9,79 (14)	11,18 (17)	**33,33 (53)**		**4,50 (2,61** – **7,76)**
Tórax	14,91 (89)	12,59 (18)	16,78 (24)	16,45 (25)	13,84 (22)		1,17 (0,64–2,14)
Miembros superiores	16,42 (98)	15,38 (22)	21,68 (31)	19,74 (30)	9,43 (15)		0,64 (0,33–1,24)
Miembros inferiores	24,46 (146)	20,28 (29)	32,17 (46)	25,66 (39)	20,13 (32)		1,00 (0,58–1,71)
Diferentes partes del cuerpo	28,31 (169)	44,06 (63)	19,58 (28)	26,97 (41)	23,27 (37)		1

Fuente: datos de la Dirección de Tránsito Municipal y la Secretaría de Salud de Bucaramanga.

p-valor: prueba chi-cuadrado de Pearson y Exacta de Fisher; OR: *odds ratio* ; (IC95%): intervalo de confianza del 95%.

Referente a las variables relacionadas con el cumplimiento de normas, se identificó que quienes portan la licencia y seguro obligatorio representan un menor porcentaje en el Q4 que en los Q1-Q3. Los OR son protectores con intervalos de confianza que no atraviesan el valor de asociación nula. Por otra parte, la gravedad reportada por la Dirección de Tránsito Municipal considera que de quienes acudieron a las IPS de Bucaramanga el 92,24% (749) fueron clasificados como lesionados, el 1,60% (13) fallecieron y el 6,16% (50) sin gravedad, los OR e IC de estas dos últimas categorías son inferiores a 1.

Respecto a la hipótesis del siniestro, no se identificaron diferencias estadísticamente significativas que afecten el costo en la comparación por cuartiles; sin embargo, cuando la hipótesis del siniestro fue embriaguez el OR fue de 12,44 (IC95% 2,01–76,87) ( Tabla 3 ).

El tipo de vehículo involucrado en los pacientes que acuden a las IPS es principalmente la motocicleta, con porcentajes mayores al 83% en todos los cuartiles; además, en el Q4 sobresale, en segundo lugar, la bicicleta con el 7,88% (16). De esta forma, los OR identificados fueron 2,79 (IC95% 1,25–6,24) y 7,66 (IC95% 2,70–21,68) al comparar con los siniestros que involucran automóvil, respectivamente.

Para la parte del cuerpo afectada, se aprecia que las lesiones en la cabeza tienen mayor proporción en el Q4 con el 33,33% (53), seguidamente las lesiones por miembros inferiores con el 20,13% (32). Si la parte del cuerpo afectada es la cabeza, el OR fue de 4,50 (IC95% 2,61–7,76) en comparación con la categoría que asume diferentes partes del cuerpo ( Tabla 3 ).

## DISCUSIÓN

Son pocos los estudios que informan las características y costos de las lesiones ocurridas por siniestros de tránsito. En Colombia, algunos estudios han descrito la incidencia, los factores asociados y las características de las lesiones ocurridas, y han reportado que los motociclistas son quienes más sufren lesiones^4 , 9^, las partes del cuerpo más afectadas son la cabeza y las extremidades^9^, el trauma craneoencefálico está asociado a la mortalidad^4 , 10^y entre los factores de riesgo se identifican el desacato a las normas de tránsito^4^y la imprudencia del conductor^10^.

En nuestro estudio, los siniestros de tránsito se presentan más en hombres, jóvenes, con licencia de conducción al día, que en su mayoría manejaron con exceso de velocidad y desobedecieron las señales o normas de tránsito, hallazgos similares a lo reportado por otros autores^11^.

En relación al tipo de vehículo involucrado, primero, se encontró el automóvil, seguido de la motocicleta^14 , 15^. Sin embargo, cuando se consideran solo los datos de quienes acudieron a instituciones de salud, el principal vehículo involucrado fue la motocicleta, al igual que en otros estudios^12 , 16^.

En cuanto a las lesiones presentadas, en nuestro estudio las principales partes afectadas fueron miembros superiores, miembros inferiores y cabeza, a diferencia del estudio en Brasil que identificó lesiones en la cabeza, sin embargo, en esta última se analizaron solo accidentes en moto^17^.

Por otra parte, la mediana de costos por siniestro de tránsito fue de US$ 56 (RI = 29,35–140,15) y el promedio 290,11 ± 731,22 (IC95% 239,74–340,48) correspondiente solo a los gastos médicos derivados de su permanencia en el hospital, podría considerarse como un costo bajo comparado con el estudio realizado en Irán donde el promedio del costo está en US$ 9,024^11^, pero se debe tener en cuenta que en nuestro estudio no se incluyeron costos indirectos, costos por pérdida de productividad derivada del accidente que, según otros estudios, pueden ser más altos que los netamente relacionados a la atención en salud debido al accidente^15 , 18 , 19^.

De esta manera, los costos derivados de los siniestros de tránsito pueden generar impacto socioeconómico a la comunidad, por ello la importancia de adoptar medidas de prevención encaminadas a reducir la ocurrencia de los mismos. Una de esas medidas puede ser la toma de acciones para mejorar la congestión vehicular, ya que algunos estudios han encontrado que volúmenes de tráfico alto pueden aumentar la probabilidad de accidentes debido a que hay más automóviles en la carretera. Sin embargo, hay que tener en cuenta que, en volúmenes de tráfico bajo, los accidentes también son frecuentes como resultado de altas velocidades; esto último sugiere intervenciones en regulaciones y controles de velocidad en las carreteras^20 , 21^.

En cuanto a este hallazgo es conveniente continuar explorando los efectos de la congestión vehicular en los accidentes de tránsito, dado que los resultados del presente estudio, como no respetar la prelación, adelantar invadiendo, adelantar por derecha y girar bruscamente, podrían suponer comportamientos derivados del afán o premura luego de pasar por un periodo de tráfico vehicular.

En nuestro estudio se encontró una relación estadísticamente significativa de los costos por accidentes de tránsito con la edad, portar licencia y seguro al día, la gravedad del accidente según la clasificación realizada por la dirección de tránsito y la parte del cuerpo afectada, similar a lo reportado en estudio llevado a cabo en Irán^11^. También, se observó relación estadísticamente significativa entre la gravedad de la lesión y el aumento de los costos similar a lo reportado en un estudio en Haití donde los pacientes lesionados en accidentes de carretera representaron el mayor costo con un valor promedio de US$ 1,220^22^.

En Argentina, Brasil, Colombia y México, entre los años 2006 y 2015 se calculó en 13.540 millones de dólares la pérdida acumulativa del PIB causada por las enfermedades crónicas no transmisibles más frecuentes, cardiopatías, los accidentes cerebrovasculares y la diabetes^23^. En Colombia, para el periodo 2008–2010 la Universidad de los Andes calculó el costo de los accidentes de tránsito alrededor del 1% del PIB, valor que contempla los costos médicos, humanos, administrativos y las pérdidas económicas por daños a la propiedad^24^. En el 2016 se estimó en 1,52 billones de pesos anuales el costo de los accidentes de tránsito asumido por el Seguro Obligatorio de Accidentes de Tránsito y la Administradora de los Recursos del Sistema General de Seguridad Social en Salud^24^.

A pesar que en nuestro estudio no se encontró relación estadísticamente significativa entre el género y los costos, sí se observó mayor número de accidentes en hombres, lo cual podría ser considerado como un factor de riesgo y enfoque de las medidas de prevención en esta población. Esta condición también se identificó en Brasil^14^donde fue asumida como un factor en el que se presenta aumento de las secuelas y, por ende, de los costos por pérdida de capacidad de producción.

Los resultados del estudio ponen de manifiesto la necesidad de centrar la prevención en los factores identificados con mayor frecuencia en este tipo de accidentes como el género masculino, la edad joven, la motocicleta como vehículo de transporte y la prevalencia de algunos comportamientos no adecuados en el momento de manejar, sin que esto se relacione con el tener la licencia y documentación de seguros en regla. De esta manera, se deben enfocar acciones educativas que generen una conciencia colectiva frente al cambio de comportamientos de riesgo para accidentarse. Es de resaltar que en varios países se están implementando una licencia de conducción por puntos^25^, como una manera de vigilar y estar reeducando a los conductores. Lo anterior permitiría reducir los gastos derivados de los accidentes de tránsito en el sistema de salud y en la comunidad que permitan derivar los recursos hacia la suplencia de otras necesidades básicas de la población y, así, probablemente reducirían la carga económica de la nación^22^.

Para el presente estudio se reconocen limitaciones como: no se obtuvo información de costos de atención de IPS de otros municipios del área metropolitana (Floridablanca, Piedecuesta y Girón); además, en la información reportada por las IPS respecto de la CIE-10, 209 registros no proporcionaban datos sobre la parte del cuerpo afectada específicamente y asignaron un CIE-10 referente al actor vial (peatón, ciclista, motociclista, caída).

También se debe tener en cuenta que los costos generados pueden ser inferiores, dado que los recursos humanos y tecnológicos para la atención en salud pueden ser menores que los de países desarrollados. De otra parte, sería interesante en estudios futuros vincular variables, como ingesta de alcohol, condiciones de las vías donde ocurrió el siniestro de tránsito, uso de elementos de proyección personal, y analizar los costos indirectos como la pérdida de la capacidad de producción por las secuelas dejadas por el accidente, de esta manera se podría brindar una proyección más amplia para las asignaciones presupuestales a estos eventos y las tarifas previstas por las aseguradoras para atender esta situación, tal como nos plantean otros estudios^19 , 26^; además, desglosar los costos de atención medica directa por profesionales, medicamentos, transporte, ayudas diagnósticas y dispositivos^15 , 19^. Adicionalmente, vale la pena indagar respecto de la calidad de las motocicletas, de los elementos de protección personal y el uso adecuado de los mismos.

Finalmente, se identificó que los factores relevantes de un aumento considerable de los costos en los accidentes de tránsito fueron la motocicleta, la bicicleta, ser menor de 18 años o tener entre los 46 y los 60 años, lesión en la cabeza y embriaguez como hipótesis. Contrariamente se encontró una disminución de los costos en los accidentes de tránsito en los casos que el conductor cumplía con la normatividad de portar licencia de conducción y póliza de seguro obligatorio de accidente. Es relevante identificar mecanismos para disminuir este tipo de eventos, siendo conocedores de que son prevenibles, se pueden implementar medidas para disminuir su severidad y, por ende, los costos y secuelas para la sociedad.
